# Impact of Artificial Sweeteners on Inflammation Markers: A Systematic Review of Animal Studies

**DOI:** 10.3390/nu17203251

**Published:** 2025-10-16

**Authors:** Pauline Celine Raoul, Maurizio Romano, Francesca Sofia Galli, Marco Cintoni, Esmeralda Capristo, Vincenzina Mora, Maria Cristina Mele, Antonio Gasbarrini, Emanuele Rinninella

**Affiliations:** 1 Clinical Nutrition Unit, Department of Medical and Abdominal Surgery and Endocrine-Metabolic Sciences, Fondazione Policlinico Universitario Agostino Gemelli IRCCS, Largo A. Gemelli 8, 00168 Rome, Italy; paulineceline.raoul@policlinicogemelli.it (P.C.R.); f.sofiagalli@gmail.com (F.S.G.); marco.cintoni@unicatt.it (M.C.); mariacristina.mele@unicatt.it (M.C.M.); 2 Degree Course in Dietetics, Università Cattolica del Sacro Cuore, Largo F. Vito 1, 00168 Rome, Italy; maurizio.romano01@icatt.it; 3 Research and Training Center in Human Nutrition, Catholic University of the Sacred Heart, Largo A. Gemelli 8, 00168 Rome, Italy; esmeralda.capristo@unicatt.it (E.C.); antonio.gasbarrini@unicatt.it (A.G.); 4 Obesity Disorders Unit, Department of Medical and Abdominal Surgery and Endocrine-Metabolic Sciences, Fondazione Policlinico Universitario Agostino Gemelli IRCCS, Largo A. Gemelli 8, 00168 Rome, Italy; 5 Digestive Disease Center (CEMAD), Department of Medical and Abdominal Surgery and Endocrine-Metabolic Sciences, Fondazione Policlinico Universitario Agostino Gemelli IRCCS, Largo A. Gemelli 8, 00168 Rome, Italy; vincenzina.mora@policlinicogemelli.it; 6 Clinical Trial Office (CTO), Fondazione Policlinico Universitario Agostino Gemelli IRCCS, Largo A. Gemelli 8, 00168 Rome, Italy; 7 Department of Translational Medicine and Surgery, Università Cattolica del Sacro Cuore, Largo F. Vito 1, 00168 Rome, Italy

**Keywords:** artificial sweeteners, inflammation, food additives, aspartame, sucralose, saccharin, acesulfame potassium, intestinal barrier, gut microbiota

## Abstract

Background: Artificial sweeteners, widely used as non-nutritive sugar substitutes, are increasingly prevalent in ultra-processed products. Although promoted for weight management due to their minimal caloric content, their impact on systemic inflammation remains uncertain. This systematic review of animal studies aims to evaluate the association between artificial sweetener consumption and inflammatory biomarkers. Methods: A systematic literature search was conducted up to May 2025 across PubMed, Web of Science, and Scopus, following PRISMA guidelines and registered in PROSPERO (CRD420251084004). Risk of bias was assessed using the ARRIVE guidelines and SCYRCLE’s risk of bias tool. Results: Thirty-seven animal studies were included: aspartame (*n* = 17), sucralose (*n* = 16), acesulfame potassium (*n* = 5), and saccharin (*n* = 4). Protocols varied in terms of dosage, exposure duration, animal models, and assessment of inflammatory outcomes, including C-reactive protein, interleukins (IL-6 and IL-1β), and tumor necrosis factor alpha. Aspartame and sucralose could elevate inflammatory markers, with sucralose also disrupting gut integrity and microbiota. Acesulfame K and saccharin showed variable, dose-dependent effects. Conclusions: This systematic review of animal studies suggests a possible mechanistic association between the consumption of certain artificial sweeteners and systemic inflammation. However, this relationship remains to be clarified and warrants exploration through well-designed, large-scale randomized controlled trials.

## 1. Introduction

Obesity and non-communicable diseases (NCDs) are becoming a critical issue for modern healthcare systems worldwide, hampering their sustainability and effectiveness. In the last decade, the prevalence of overweight and obesity increased dramatically, reaching 39% globally in 2023, under the effect of the westernization of the diet, the excessive food intake—including palatable and high-calorie foods, ultra-processed foods (UPFs)- and a sedentary lifestyle [1]. On the other hand, non-nutritive sweeteners (NNS) have become increasingly prevalent in modern diets due to their use as sugar substitutes in foods, beverages, and pharmaceuticals [2]. Promoted for their negligible caloric value, artificial sweeteners are often recommended for weight management and sugar reduction [3]. To date, global per capita consumption of beverages containing NNS has increased by 36% [2]. The increasing use of NNS to sweeten drinks and packaged food in upper-middle-income countries and lower-middle-income countries [2] has garnered growing attention in public health nutrition policymaking and the World Health Organization (WHO) [4]. The WHO finds no safety signals associated with NNS use in randomized controlled trials (RCTs), although observational studies raise concerns; EFSA’s reevaluations have upheld safety and sometimes increased acceptable daily intake (ADI) of some NNS. In recent years, researchers have increasingly focused attention on the long-term biological effects of these substances—particularly their influence on health [5,6]. Chronic low-grade inflammation is a well-established contributor to the pathogenesis of metabolic disorders, including obesity, insulin resistance, cardiovascular disease, and type 2 diabetes [7]. The WHO-supported systematic review and meta-analysis of 2022 by Rios-Leyvraz et al. found that in short-term RCTs, NNS led to lower body weight, body mass index (BMI), and energy intake—especially when compared to sugars, but not to water [8]. Although its design is prospective, the NutriNet-Santé cohort, published in 2023, comprising 105,588 participants followed for a median of 9.1 years, revealed a higher risk of developing type 2 diabetes among individuals who consumed artificial NNS—specifically, aspartame (ASP), acesulfame potassium (Ace-K), and sucralose (SUC)—compared with those who did not [9]. A recent 12-week, parallel-arm RCT of 210 participants with type 2 diabetes assessed no significant differences in terms of C-reactive protein (CRP) and tumour necrosis factor alpha (TNF-alpha) levels between the intervention group, where sugar/sucrose in coffee or tea was substituted with sucralose and the control group where sugar/sucrose was continued [10]. The SWEET project Health Impact Database was recently developed as the first of its kind, systematically gathering data from human clinical studies on various NNS conducted between 2000 and 2024 [11]. This project will be a valuable tool for researchers, enabling them to perform systematic reviews and meta-analyses of RCTs. In terms of health outcomes, body weight, glucose homeostasis, and energy intake were the most extensively studied. In contrast, oral health and the gut microbiome received comparatively less attention [11]. On the other hand, the consumption of artificial NNS has been linked to gut dysbiosis, which could potentially disrupt metabolic signalling pathways and immune responses [12], exacerbating gut inflammation and increasing disease susceptibility [13]. Otherwise, a recent multicentre RCT of adults with overweight or obesity observed in adults consuming NNS compared with those who did not (sugar group) an improved weight loss maintenance accompanied by altered gut microbiota composition, with a higher abundance of SCFA-producing and CH4-producing bacterial taxa [14]. These findings underscore the need for a nuanced understanding of the dual effects of artificial NNS on health. Numerous studies in animal models have been performed to assess the impact of artificial sweeteners on inflammatory biomarkers, such as CRP, interleukins (e.g., IL-6, IL-1β), and TNF-α [15,16,17].

This systematic review aims to synthesize and critically evaluate the existing body of evidence from animal studies examining the influence of artificial NNS on inflammation-related biomarkers.

## 2. Methods

This systematic review is structured following the general principles published in the Preferred Reporting Items for Systematic Reviews and Meta-Analyses (PRISMA) guidelines [18]. The PRISMA checklist was detailed in Table S1. Full details of the search strategies were specified and documented in a protocol that was registered at PROSPERO (https://www.crd.york.ac.uk/PROSPERO, accessed on 15 July 2025) as ID CRD420251084004.

### 2.1. Eligibility Criteria

The eligibility criteria are outlined using the PICOS format (Table 1).

The exclusion criteria were as follows: (1) in vitro or human studies; (2) reviews; and (3) studies not fulfilling the inclusion criteria.

### 2.2. Data Sources and Search Strategy

The search was carried out on 5th May 2025 using three electronic databases, MEDLINE (via PubMed), ISI Web of Science, and Scopus. The search string for each database is described in Table S2. A manual search of eligible studies has been conducted to identify studies that may not have been identified in the databases.

### 2.3. Study Selection

The study selection process was independently conducted by two reviewers (M.R. and P.R.) following standardized eligibility criteria. All articles retrieved through the electronic search were imported into Mendeley© reference management software (Elsevier, Amsterdam, The Netherlands, Version 1.19.5), where duplicates were automatically and manually identified and removed. Subsequently, the reviewers independently screened the titles and abstracts of the remaining studies to determine their eligibility. Studies that did not meet the predefined inclusion criteria were excluded at this stage. Full-text articles were then obtained for references deemed potentially eligible, and a second round of screening was performed to ensure compliance with inclusion and exclusion parameters. Any instances of disagreement or discrepancy between the reviewers were resolved through collaborative discussion and consensus.

### 2.4. Data Extraction and Reporting

After full-text analysis, the following information was extracted from the included articles: title, author information, year of publication, type of study performed, assessed outcome/s, the animal model used, animal gender, age, and weight at baseline, administered dose, length of study, administration route, and main conclusions. Data were reported using an Excel spreadsheet (Microsoft Office, Redmont, WA, USA) developed explicitly for this study. Each full-text article was retrieved, and any ineligible articles were excluded from the reasoning reported. Disagreements between the two independent reviewers (M.R. and P.R.) were resolved by discussion using pre-specified selection and quality criteria, and persistent disagreements were adjudicated by a third independent reviewer (E.R.).

### 2.5. Quality Assessment

The quality of the included studies was assessed following the Animal Research Reporting of In Vivo Experiments (ARRIVE) guidelines [19]. These guidelines consist of the minimum information that animal research studies should include, such as the number and specific characteristics of animals, details of housing and husbandry, experimental and statistical methods, and reporting and interpretation of the results. Moreover, the SCYRCLE’s risk of bias tool [20] was used to assess the risk of bias of animal studies. The SCYRCLE’s tool is an adapted version of the Risk of Bias tool provided by the Cochrane Collaboration. It consists of ten entries associated with selection bias, performance bias, detection bias, attrition bias, reporting bias, and other biases. Quality assessment was independently performed by two reviewers (S.G. and P.R.), and a consensus should be reached for discrepancies.

## 3. Results

### 3.1. Study Selection

**Figure 1:**
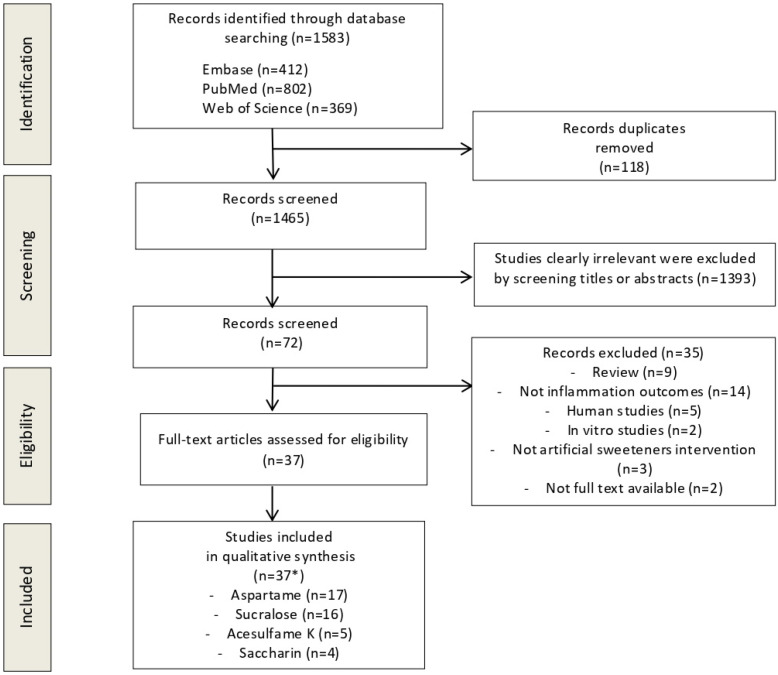
Preferred reporting items for systematic reviews and meta-analyses (PRISMA) flow diagram. Abbreviations: *, some studies studied more than one sweetener; K, potassium.

The flow diagram in Figure 1 displays the results of the literature search and study selection process. A total of 1583 studies were initially identified. After duplicate removal (*n* = 118), a total of 1435 studies remained for title and abstract screening. A total of 35 studies were excluded for the following reasons: human studies (*n* = 5), in vitro studies (*n* = 2), no assessment of outcomes of interest (*n* = 14), not artificial sweetener interventions (*n* = 3), reviews (*n* = 9), not full-text available (*n* = 2). Finally, 37 studies were identified for inclusion in the systematic review and included in the qualitative synthesis.

### 3.2. Study Characteristics

The characteristics of each study included are detailed in Table 2. All studies [5,6,15,16,17,21,22,23,24,25,26,27,28,29,30,31,32,33,34,35,36,37,38,39,40,41,42,43,44,45,46,47,48,49,50,51,52] were conducted between 2012 [21] and 2025 [37], drawing primarily from investigations in rodents, with rare inclusions of non-mammalian models like zebrafish [23] and non-human primates [24]. As regards mice, the C57BL/6 strain was used in more than half of the studies [5,16,17,23,25,26,27,28,29,30,31,32,33,34,35,45]—followed by Wistar and Sprague Dawley rats. Among the 41 studies included, 26 used exclusively male animals. Eight studies used exclusively female animals [5,7,8,9,11,16,23,31]. One study included both sexes [48]. In four studies, the sex of the animals was not reported [19,22,25,27]. Most animals ranged from 3 weeks [10] to 6 months of age [27], representing both juvenile and adult phases, with body weights recorded between 17 g [36] and 250 g [42]. A diverse array of artificial sweeteners was studied. ASP emerged as the most frequently studied compound (*n* = 17), followed closely by sucralose (SUC) (*n* = 16), and to a lesser extent, ACK (*n* = 5), saccharin (SAC) (*n* = 4). Across the included studies, the approximate dosage range of sweeteners varied widely, from as low as 0.625 mg/kg [21] to as high as 2000 mg/kg [33]. Administration routes were predominantly oral (gavage or drinking water), though subcutaneous and topical methods were also used.

### 3.3. Quality Assessment

The comparative assessment of compliance with the ARRIVE 2.0 guidelines across the included in vivo studies revealed several recurring weaknesses in reporting quality, particularly concerning methodological transparency and animal welfare practices (see Supplementary Table S3). Although most studies clearly described their experimental design, outlined group allocations, and reported key outcome measures, important methodological aspects were frequently underreported. Notably, while sample sizes were consistently stated, very few studies justified their group sizes through a priori sample size calculations, limiting confidence in the statistical power and raising concerns regarding unnecessary animal use. Randomisation was occasionally mentioned, but often without specifying the method employed, and information on blinding was largely missing. Another limitation was the lack of detailed inclusion and, especially, exclusion criteria. In nearly all studies, there was no indication of whether any animals or data points were excluded from analysis, nor how such decisions were made. Criteria for animal care and monitoring were also insufficiently reported: few studies described procedures implemented to minimise pain, distress, or humane endpoints. Regarding housing and husbandry, general environmental conditions such as temperature and light/dark cycles were frequently stated; however, information about enrichment strategies was mostly absent. Furthermore, almost none of the studies indicated that the research protocol had been pre-registered. Despite these limitations, several ARRIVE criteria were generally well addressed across studies. Abstracts were typically complete and informative background sections offered clear scientific justification, objectives were stated explicitly, and outcome measures and statistical methods were usually appropriate and adequately described. Ethical approval was also consistently reported. However, the overall picture suggests that, while baseline reporting practices are in place, key elements related to methodological rigour and animal welfare are often not reported consistently. Improving the quality and completeness of reporting, especially regarding aspects such as randomisation, blinding, exclusion criteria, and animal care, would significantly enhance the reproducibility and reliability of preclinical animal research.

The methodological quality of the included animal studies was assessed using SYRCLE’s Risk of Bias tool (see Supplementary Table S4). Overall, most studies presented a moderate to high risk of bias, particularly in domains related to sequence generation, allocation concealment, random housing, blinding of caregivers and outcome assessors, and random outcome assessment. Randomization was often mentioned, but in many cases, the method used to generate the random sequence was not reported, making it difficult to assess whether it was applied appropriately. Similarly, allocation concealment procedures were either unclear or not described. Blinding of caregivers and outcome assessors was rarely implemented or explicitly stated, and random selection of animals for outcome assessment was generally not reported. In contrast, the domains related to baseline group characteristics, completeness of outcome data, selective outcome reporting, and other sources of bias were generally well addressed across studies, with low risk of bias observed.

### 3.4. Results

#### 3.4.1. Aspartame (17 Studies)

Table 3 presents findings from 17 in vivo animal studies on ASP consumption and inflammation markers. These studies varied in animal models, ASP dosages (17 mg/kg to 2000 mg/kg), and treatment durations, assessing markers such as pro-inflammatory cytokines (e.g., TNF-α, IL-1β, IL-6), anti-inflammatory cytokines (e.g., IL-4, IL-10), chemokines—monocyte chemoattractant protein-1 (MCP-1)—and inflammatory mediators—e.g., Nuclear Factor kappa-light-chain-enhancer of activated B cells (NF-κB), inducible nitric oxide synthase (iNOS), prostaglandin E2 (PGE2). ASP exposure significantly increased pro-inflammatory cytokines, including TNF-α, IL-6, and IL-1β, in various tissues such as blood serum [16,27], prefrontal cortex, hippocampus [17,18,38], colon, liver, and adipose tissue [28,30,45]. Also, one study reported that ASP consumption in mice with high-fat diet-induced obesity was associated with increased inflammatory macrophage infiltration in fat tissues and elevated pro-inflammatory cytokine release [45]. Zhong et al. show that, even at ADI levels, ASP can worsen enteritis pathology and systemic inflammation [30]. Long-term or maternal ASP exposure was associated with altered T-helper cytokine profiles. Perinatal exposure models demonstrated increased Th2-associated cytokines and IL-17A in offspring, accompanied by reduced interferon (IFN)-γ expression. Maternal ASP exposure also resulted in changes to the Th1/Th2 cytokine balance in offspring through nuclear factor-κB activation [22,37]. Elevated pro-inflammatory mediators were observed concurrently with oxidative stress and neuroendocrine dysregulation in specific models [40]. These included increased circulating corticosterone levels and altered redox balance. Studies reported an association between ASP exposure and cerebral cortex injury, characterized by oxidative stress, inflammation, mitochondrial dysfunction, and apoptosis in both in vitro and in vivo models [29]. ASP exposure was also found to increase corticosterone levels and lipid peroxidation, with concurrent observations of oxidative stress and altered cytokine secretion [40]. ASP exposure resulted in neuroinflammation, including microglial activation and increased pro-inflammatory cytokine production, even at low concentrations [45]. Conversely, other studies employing shorter exposure durations or lower doses did not detect significant changes in IFN-γ, TNF-α, IL-1β, IL-6, IL-2, or IL-4 levels [33,46]. He et al. reported an increase in anti-inflammatory mediators, specifically transforming growth factor-beta (TGF-β) and IL-10, following ASP exposure [42]. ASP exposure modulated chemokine expression, specifically CCL2/MCP-1 and CXCL10, as well as their receptors. CX3CL1 was identified as the most upregulated gene in ASP-fed mice, with significantly elevated circulating protein levels compared to controls [24,28]. Alterations in immune cell populations were reported following ASP exposure. In vivo studies showed increased infiltration of CD68^+^ macrophages and Iba-1^+^ microglia in both brain and adipose tissues, accompanied by morphological changes [32,45]. Intracellular signaling pathways related to inflammation and immune activation were affected by ASP. NF-κB activation was consistently reported, evidenced by increased nuclear translocation of p65 and upregulation of genes such as TNF-α, iNOS, and COX-2. Multiple studies observed elevated NF-κB activation and reduced IκB expression in brain, lung, and liver tissues, with increased expression of mediators including iNOS, nNOS, caspase 8, and JNK3 [37,38]. Evidence from hepatic tissue also demonstrated NLRP3 inflammasome activation and increased levels of cleaved caspase-1. Additionally, long-term ASP intake was associated with increased expression of stress-response proteins and markers of apoptosis [38].

#### 3.4.2. Sucralose (16 Studies)

Table 4 presents the results of in vivo animal studies examining the effect of SUC consumption on inflammatory markers. Findings from 16 studies consistently show a significant association between SUC consumption and indicators of inflammation, observed across various experimental designs, dosages, and exposure durations. SUC exposure has been found to affect inflammatory markers locally and systemically.

These studies utilized diverse animal models, with SUC administered at dosages ranging from dietary-relevant to high pharmacological levels over periods of 4 weeks to 6 months. Across these models, SUC exposure was associated with the upregulation of pro-inflammatory cytokines, reduction in anti-inflammatory mediators, and activation of inflammatory pathways.

Consistently elevated levels of TNF-α, IL-6, and IL-1β were observed across various tissues, including the colon, liver, and Peyer’s patches [17,25,36,39,47]. These cytokine increases were frequently accompanied by the upregulation of Toll-like receptors and activation of the NF-κB pathway [17,36,48,49]. Research also demonstrated that SUC consumption was associated with suppressed anti-inflammatory mediators, including a notable reduction in IL-10 and IκBα [15,17,39]. Additionally, elevated levels of IL-17 and IL-12 were reported in the gut-associated lymphoid tissue. These findings were observed with chronic SUC consumption or in conjunction with a high-fat diet [25,44]. Increased circulating lipopolysaccharides (LPS) and reduced levels of occludin and secretory IgA were observed. These alterations were accompanied by elevated expression of TLR4 and NF-κB [36,49]. Regarding the gut microbiota, chronic intake of SUC was associated with decreased bacterial diversity and enrichment of pro-inflammatory microbial gene profiles. These microbial shifts occurred alongside increased IL-17, IL-12, and CD19^+^ B cells [5,25,44]. Evidence suggests that artificial sweeteners, including SUC and SAC-cyclamate mixtures, are associated with systemic inflammatory effects that extend beyond the gut. Chronic consumption has been shown to elevate circulating levels of IL-6, TNF-α, and LPS [36,47]. These changes correlated with histopathological alterations observed in the liver, kidney, pancreas, and urinary bladder, including inflammatory infiltrates and tissue damage [36]. Within the liver, SUC exposure increased iNOS and MMP-2. Furthermore, SUC consumption exacerbated hepatic steatosis and inflammatory cytokine expression in models of diet-induced obesity [47].

#### 3.4.3. Acesulfame-K (5 Studies)

Table 5 summarizes five in vivo studies evaluating the effects of Ace-K on inflammation-related markers in animal models. The studies vary in species, exposure duration, doses, and assessed outcomes. In the study by Zhai et al. [43], C57BL/6 mice and zebrafish were exposed to different concentrations of Ace-K (ranging from 10 µg/L to 21 mg/L) for 21 to 28 days. The results indicated an increased infiltration of inflammatory cells in colonic tissue and a reduction in protective mucin secretion, compared to water controls. Histological analysis revealed epithelial and crypt damage, mucus depletion, and signs of inflammation. Transcriptomic analyses showed increased expression of genes related to cytokine–cytokine receptor interaction, chemokine signaling, and pathways associated with inflammatory bowel disease. Bridge-Comer et al. [41] investigated the effects of maternal Ace-K intake (12.5 mM in drinking water) during gestation and lactation in C57BL/6 mice. In adult offspring, increased TNF-α mRNA expression was observed in the skin of females compared to both water and fructose controls. No significant variation was detected in males. Other inflammation-related markers, including IL-1β, Nlrp3, Vascular endothelial growth factor (VEGF) A, TGF-β, and PPARγ, showed no significant differences between the sexes.

In the study by Lin et al. [22], ApoE-/- mice were exposed to Ace-K (15 mg/kg/day) for 8 weeks. No significant differences were found in the expression of TNF-α, Ccl2, or IL-6 (mRNA) in treated animals compared to saline controls. Hanawa et al. [35] administered Ace-K (150 mg/kg/day) to C57BL/6J mice for 8 weeks. An increase in the expression of TNF-α, IFN-γ, IL-1β, and MAdCAM-1 (mRNA) was observed in the small intestinal mucosa when compared to water-treated controls. Finally, in Shou et al. [52], C57BL/6 mice were treated with Ace-K at two doses (40 and 120 mg/kg/day) via oral gavage for 11 weeks. In the high-dose group, increased levels of TNF-α, IL-6, and LPS were reported compared to water controls. In the low-dose group, IL-6 levels were elevated, whereas IL-1β levels did not differ significantly from those of the control group [52].

#### 3.4.4. Saccharin (4 Studies)

Table 6 presents the results of in vivo RCTs examining the effect of saccharin (SAC) consumption on inflammatory markers in murine models. The studies differ in terms of animal strain, duration of exposure, dosage, and outcome measures. In the study by Zhang et al. [34], C57BL/6 mice were treated with SAC (5 mg/kg/day) for 28 days in a DSS-induced colitis model. At the end of the treatment, an increase in IL-6, IL-17A, and TNF-α (mRNA) levels was observed in the SAC + DSS group compared to the water control. A decrease in the same markers was observed when comparing the SAC + DSS group to the DSS-only group. Hanawa et al. [35] conducted an 8-week study using C57BL/6J mice exposed to SAC at 50 and 150 mg/kg/day. The analysis of TNF-α, IFN-γ, and IL-1β did not reveal significant differences between the treated animals and the controls. No substantial changes in histological markers of intestinal inflammation were reported. In the study by Bian et al. [6], C57BL/6J mice received SAC (0.3 mg/mL in drinking water) for a duration of six months. An increase in hepatic iNOS and TNF-α mRNA expression was observed in SAC-treated animals compared to controls. No significant differences were noted for IL-6 and IL-1β levels. Finally, Madboury et al. [36] evaluated the effects of SAC-cyclamate in BALB/c mice over 8 and 16 weeks. At 16 weeks, an increase in IL-6 was detected in SAC-treated animals compared to the water control. Circulating LPS levels were also elevated in SAC-treated groups. TNF-α levels were higher in the SUC group than in the SAC-cyclamate group at both timepoints.

## 4. Discussion

Due to their strong sweetening power per gram compared to sugars and the absence of caloric intake, artificial NNS are commonly used to replace added sugar in beverages, chewing gum, food, and pastries. However, the ubiquitous presence of artificial sweeteners in the global food market for human use necessitates a thorough understanding of their long-term biological effects, particularly concerning systemic inflammation. This systematic review aimed to synthesize evidence from animal studies to clarify the impact of artificial NNS on inflammation-related biomarkers, offering critical insights into this controversial topic. Overall, this systematic review suggests a potential complex relationship between the consumption of some artificial sweeteners and inflammatory responses. The observed effects are notably varied, influenced by the specific sweetener, dosage, duration of exposure, and the animal model used.

ASP is a synthetic, non-nutritive sweetener that has been widely used in food and pharmaceuticals, including diet drinks, gum, desserts, dairy products, cereals, toothpaste, and chewable medications. The International Agency for Research on Cancer (IARC), in collaboration with the WHO, and the Joint FAO/WHO Expert Committee on Food Additives (JECFA) classified ASP as *possibly carcinogenic to humans* (Group 2B), citing limited evidence for carcinogenicity in humans. In parallel, JECFA affirmed the ADI of 40 mg/kg body weight, concluding that ASP consumption within this threshold does not pose a health risk [53]. For ASP, evidence from 17 studies indicates a multifaceted interaction with inflammatory markers. Exposure consistently led to significant increases in pro-inflammatory cytokines such as TNF-α, IL-6, and IL-1β across various tissues, including blood serum, specific brain regions (prefrontal cortex, hippocampus), colon, liver, and adipose tissue. These increases were often dose (starting from 17 mg/kg)- and time-dependent. ASP was also shown to exacerbate high-fat diet-induced obesity, promoting inflammatory macrophage infiltration and cytokine release. Concerns extend even to ADI levels, where ASP could worsen enteritis pathology and systemic inflammation. Mechanistically, ASP is associated with oxidative stress and neuroendocrine dysregulation, characterized by increased circulating corticosterone and lipid peroxidation [22]. The activation of NF-κB and the NLRP3 inflammasome was a consistent finding, leading to increased expression of NF-κB-dependent genes, such as TNF-α, iNOS, and COX-2 [2,6]. While some studies with shorter durations or lower doses did not detect significant changes, a compensatory increase in anti-inflammatory mediators, such as TGF-β and IL-10, was occasionally observed [22,27,28]. Neuroinflammation and hypothalamic inflammation are pathological features of a dysregulation of the homeostatic and hedonic mechanisms of food intake and, consequently, have been recently associated with compulsive/addictive feeding in mouse models [54].

SUC is a chemically synthesized sweetener derived from sucrose, commonly found in a wide range of carbonated beverages, tabletop sugar substitutes, salad dressings, baking mixes, and breakfast cereals. Sucralose tolerance was studied in human volunteers and was well-tolerated in single doses of up to 10 mg/kg/day and repeated doses increasing to 5 mg/kg/day (up to 500 mg/kg/day) for 13 weeks [55]. However, ADI level for SUC was set at 5 mg/kg body weight per day (mg/kg/d) in the United States and 15 mg/kg/d in the EU as recommended by the Scientific Committee on Food of the European Commission [56]. The analysis of 16 studies on SUC consistently demonstrated a pro-inflammatory footprint. SUC exposure was associated with elevated pro-inflammatory cytokines (TNF-α, IL-1β, IL-6) and a concomitant decrease in anti-inflammatory mediators (IL-10, IκBα). A critical finding was the negative impact on intestinal barrier integrity and microbial composition. This disruption was evidenced by increased circulating LPS and reduced levels of occludin and secretory IgA, indicating compromised epithelial permeability and mucosal immunity. Recent evidence confirmed a causal role of LPS in neuroinflammation and in the dysregulation of the reward system following exposure to a high-fat diet [54]. Chronic SUC intake could also be associated with reduced bacterial diversity and an enrichment of pro-inflammatory microbial gene profiles, a hallmark of obesity, metabolic, and inflammatory diseases [57]. Moreover, SUC, particularly when combined with SAC-cyclamate, exerted systemic inflammatory effects beyond the gut, with increases in circulating IL-6, TNF-α, and LPS, correlating with histopathological alterations in the liver, kidney, pancreas, and urinary bladder [57].

Ace-K exhibits a sweetness that is frequently combined with other high-intensity sweeteners to enhance palatability and improve stability. Its applications encompass a wide range of consumables, including carbonated soft drinks, frozen confections, non-alcoholic beverages, chewing gum, condiments and sauces, and fermented dairy products (e.g., yogurt). The EFSA has established an ADI of 15 mg/kg body weight/day. This value was derived from the no-observed-adverse-effect level identified in chronic toxicity and carcinogenicity assays conducted in rodents [58]. Our research identified five studies, which revealed dose- and model-dependent responses [26,35,41,43,52]. The relationship between Ace-K and inflammation is complex and context-dependent. Animal studies suggest that Ace-K can modulate inflammatory pathways, particularly in the gut. Zhai et al. [43] demonstrated intestinal inflammation and focal adhesion pathway downregulation, resembling IBD-like changes. Hanawa et al. [35] confirmed elevated pro-inflammatory cytokines and increased intestinal permeability, implicating barrier dysfunction. Together, these findings highlight the gut as a central target of Ace-K ’s immunological effects. Developmental exposure also appears critical. Bridge-Comer et al. [41] showed that maternal Ace-K intake altered the offspring’s inflammatory gene expression. Female offspring displayed heightened TNF-α responses, suggesting sex-specific vulnerability. However, Lin et al. [26] reported no systemic inflammatory activation in an atherogenic model despite lipid dysregulation. This suggests that Ace-K does not universally promote inflammation, but instead acts as a context-dependent modulator. The demonstrated effects vary with dosage. Indeed, Shou et al. [52] found significant cytokine elevation only at higher exposure levels. This aligns with threshold-like responses seen in other models. Collectively, evidence points toward localized gut inflammation and immune activation as consistent outcomes. Systemic inflammatory changes are less robust and appear model-specific. However, potential risks may be greater with chronic, high-dose, or developmental exposures. For this reason, such findings warrant further investigation into long-term metabolic and inflammatory consequences of Ace-K in RCTs.

For over a century, SAC has been used as a low-calorie sweetener in various foods and beverages. EFSA has assessed SAC and confirmed its suitability for human consumption, raising the ADI from 5 to 9 mg per kilogram of body weight. The previous limit, set in 1995, reflected findings of a higher rate of bladder tumors in experimental rat models. Current evidence, however, indicates that these effects are confined to male rats and do not apply to humans [59]. Results for SAC from four studies were mixed [5,34,35,36]. One study reported SAC attenuated DSS-induced colitis by reducing colonic pro-inflammatory cytokines [34]. Conversely, a long-term study noted inflammatory effects in the liver, with increased hepatic iNOS and TNF-α, potentially mediated by gut microbiota alterations [5]. SAC-cyclamate mixtures were also associated with increased IL-6 and circulating LPS.

This systematic review underscores critical limitations in the methodological quality and reporting of the included animal studies. A predominant issue was the moderate to high risk of bias across most studies, particularly in domains such as sequence generation, allocation concealment, and blinding of personnel and outcome assessors, which were largely unreported or unclear. Key methodological aspects, such as a priori sample size calculations, detailed inclusion/exclusion criteria, and comprehensive animal welfare practices, were frequently underreported. These shortcomings significantly diminish confidence in the statistical power, reproducibility, and overall reliability of the preclinical animal research. Improving the quality and completeness of reporting is crucial to enhancing the robustness of future studies. Moreover, the heterogeneity of time exposure, animal models, NNS type, and dosage does not permit the performance of a meta-analysis or the measurement of effect size, making it difficult to reach clear conclusions.

In humans, a recent cross-sectional observational study enrolling 624 adults in the American Cancer Society’s Cancer Prevention Study-3 Diet Assessment Substudy found a positive association between the consumption of artificial NNSs, including ASP, SAC, SUC, and Ace-K, and leptin, CRP, and IL-6. People who consume higher amounts of artificial NNS also have a lower diet quality score compared to non-consumers or low consumers, as a recent population study highlights [60]. Another more recent large prospective study, observing for 8 years a cohort of more than 12,000 participants in Brazil (mean age 51.9 ± 9.0 years), concluded that a higher consumption of artificial NNS (including ASP, SAC, ACK) was associated with a faster cognitive decline, particularly in memory and verbal fluency domains [59]. Observational studies identify long-term associations and correlations, while RCTs, although typically shorter in duration, provide stronger evidence for causality and are more informative for assessing the safety and efficacy of artificial NNS [11]. Previous meta-analyses of short-term RCTs demonstrated that low/no-calorie sweeteners may have modest benefits on measures of obesity (body weight, BMI, fat mass, and waist circumference) [61,62] but do not provide clear conclusions on inflammation markers and inflammation-associated gut microbiota variations.

A crucial issue regarding artificial NNS is the safe dose. While the regulatory agencies in healthcare set an ADI for each of them, the exact amount an individual assumes daily is not easily calculated. The quantity of artificial NNS is not clearly indicated in the food labels, unlike natural sugars; thus, the exact daily amount of artificial NNS in a typical Western diet is not so easy to ascertain. Although robust exposure assessments indicate that exceeding the ADI is uncommon in realistic scenarios [63], hypothetical intake scenarios indicate that children, because of lower body weight, are most likely to approach or exceed ADIs, warranting further research and public-health measures to monitor and limit exposure [64].

## 5. Conclusions

The WHO finds no safety signals associated with NNS use in RCTs, although observational studies raise concerns; EFSA’s reevaluations have upheld safety and sometimes increasing ADIs. This systematic review of animal studies, however, reveals that several artificial sweeteners can consistently modulate inflammatory pathways, with ASP, SUC, and Ace-K exhibiting the most robust pro-inflammatory patterns across various tissues. Effects were frequently dose- and time-dependent, and included increases in pro-inflammatory cytokines, oxidative stress, gut barrier disruption, and endotoxemia for prolonged or high-dose exposures, as well as for models combining sweetener intake with metabolic stressors, such as high-fat diets. Although animal studies identify mechanisms linking artificial sweeteners to inflammation, uncertainties in species biology, microbiota composition, dose translation, and exposure scenarios constrain their applicability to human health. Therefore, further large RCTs are necessary to better characterize the impact of artificial sweeteners on human inflammation.

## Figures and Tables

**Table 1:** PICOS criteria for inclusion of studies.

Criteria	Definition
Participants	Animals (e.g., rodents or primates) are used in controlled laboratory experiments
Exposure	Any artificial sweeteners, oral consumption: ASP, SUC, saccharin, Ace-K, neotame, or advantame
Comparator	Any alternative intervention: any other type of caloric or non-caloric sweetener, any sugar, placebo, or plain water
Outcomes	Between-group variations in inflammation-related biomarkers: - CRP in mg/L - IL-6, IL-8, IL-1β in pg/mL - TNF-α in pg/mL
Study design	All animal studies

Abbreviations: Ace-K, acesulfame potassium; ASP, aspartame; CRP, C-reactive protein; IL, interleukin; pg, picogram; mg; milligram; mL, milliliter; SUC, sucralose; TNF-α, tumour necrosis factor-alpha.

**Table 2:** Characteristics of included animal studies (in alphabetical order).

First Author, Year of Publication	Animal Type	Animal Sex	Animal Weight	Animal	Studied Artificial Sweeteners
Abdel-Salam, 2012 [21]	Wistar albino rats	male	20–22 g	n.r.	ASP
Ashok, 2015 [38]	Wistar strain Albino rats	male	200–220 g	n.r.	ASP
Babatunde, 2024 [32]	C57BL/6 Mice	male	180–230 g	n.r.	ASP
Bian, 2017 [5]	C57BL/6 Mice	male	23 g	8 weeks old	SAC
Bian, 2017 [6]	CD-1 Mice	male	n.r.	8 weeks old	SUC
Bridge-Comer, 2023 [41]	Sprague Dawley rats	female	n.r.	10 weeks old	Ace-K
Choudhary, 2015 [40]	Wistar rats	male	200–220 g	n.r.	ASP
Chuang, 2025 [37]	Wistar albino rats	female	25 g	n.r.	ASP
Dai, 2020 [47]	BALB/c Mice	female	n.r.	8 weeks old	SUC
Dai, 2021 [49]	ApoE-/- Mice	female	n.r.	8 weeks old	SUC
Escoto, 2021 [44]	BALB/c Albino Mice	male	n.r.	3 weeks old	SUC
Farahi, 2025 [33]	C57BL/6J (mice)	female	19–21 g	6–8-week-old	ASP
Farid, 2020 [48]	Swiss Mice	male, female	18–20 g	6 weeks old	SUC
Finamor, 2021 [28]	C57BL/6J Mice	male	30 g	3 months old	ASP
Guo, 2021 [17]	C57BL/6 Mice	male	20–25 g	n.r.	SUC
Hanawa, 2021 [35]	C57BL/6 Mice	male	n.r.	8 weeks old	Ace-K, SAC, SUC
He, 2023 [42]	Sprague Dawley rats	female	230–250 g	9–10 weeks old	ASP
Lawal, 2025 [22]	Wistar rats	male	80–100 g	5 weeks old	ASP
Lebda, 2017 [31]	C57BL/6J Mice	male	187.67 ± 15.14 g	6–8 weeks old	ASP
Li, 2020 [39]	Wistar rats	n.r.	n.r.	4 weeks old	SUC
Lin, 2021 [26]	C57BL/6 Mice	male	n.r.	8 weeks old	Ace-K
Liu, 2024 [46]	BALB/c Mice	male	n.r.	n.r.	ASP
Lü, 2022 [45]	C57BL/6J Mice	n.r.	n.r.	6 weeks old	ASP
Ma, 2024 [27]	C57BL/6 Mice	male	21.55–21.65 g	6 months old	ASP
Madbouly, 2022 [36]	Wistar rats	male	17–20 g	4–6 weeks old	SUC, SAC
Martínez-Carrillo, 2019 [25]	C57BL/6 mice	n.r.	n.r.	21 days old	SUC
Mohammed, 2024 [16]	C57BL/6 Mice	male	180 ± 20 g	2 months	ASP, SUC
Rosales-Gómez, 2018 [15]	Wistar rats	n.r.	n.r.	21 days old	SUC
Sánchez-Tapia, 2019 [51]	72 CD1 Mice	male	n.r.	5 weeks old	SUC
Sánchez-Tapia, 2020 [50]	BALB/c albino mice	male	n.r.	5 weeks old	SUC
Shou, 2024 [52]	Swiss albino Mice	male	n.r.	8 weeks old	Ace-K
U-pathi, 2024 [29]	C57BL/6 Mice	female	120–140 g	7 weeks old	ASP
Wu, 2025 [24]	Mice and Monkeys	male	n.r.	6–8 weeks	ASP
Yang, 2024 [23]	C57BL/6 Mice, zebrafish	female	n.r.	8 weeks old	SUC
Zhai, 2024 [43]	Sprague Dawley Rats	male	22–25 g	8 weeks old	Ace-K
Zhang, 2022 [34]	C57Bl/6 Mice	male	18–22 g	6–8 weeks old	SUC, SAC
Zhong, 2024 [30]	C57BL/6J Mice	male	n.r.	8 weeks old	ASP

Abbreviations: ASP, Aspartame; SUC, Sucralose; Ace-K, Acesulfame potassium; SAC, Saccharin; g, gram; n.r., not reported.

**Table 3:** Results of in vivo Studies assessing the impact of ASP consumption on inflammation markers (by chronological order).

Study	Animal Type	Sample Size	Time Exposure	Dose Exposure	Control Type	Outcomes	Significant * Variations of Inflammation Markers Between the Initiation and Final Timepoints	Non-Significant Variations of Inflammation Markers Between the Initiation and Final Timepoints
Abdel-Salam, 2012 [21]	Swiss albino Mice	*N* = 60 (6 × 10 groups)	4 h	0.625, 1.875, 5.625, 11.25, 22.5, and 45 mg/kg	Saline, LPS	TNF-α	At 22.5 and 45 mg/kg: ↑ TNF-α (+16.7% and +44%) vs. saline	TNF-α (ASP + LPS vs. LPS) at 0.625, 1.875, 5.625, 11.25, 22.5 mg/kg
Ashok, 2015 [38]	Wistar albino rats	*N* = 18 (6/6/6)	90 days	40 mg/kg	Saline, MTX	iNOS c-fos TNF-α Hsp70 JNK3 NFkB	↑ iNOS, c-fos, Hsp70, TNF-α, JNK3, NF-κB (mRNA) in ASP + MTX vs. saline and MTX ↑ iNOS, c-fos, Hsp70, JNK3 in ASP + MTX vs. saline and MTX	
Choudhary, 2015 [40]	Wistar albino rats	*N* = 48 (6 × 8 groups)	90 days	40 mg/kg	Saline	TNF-α IL-2 IL-4 IFN-γ	↓ IL-2, TNF-α, IFN-γ vs. saline ↑ IL-4 vs. saline	
Lebda, 2017 [31]	Wistar strain albino rats	*N* = 30 (10/10/10)	2 months	240 mg/kg	Water, soft drink	Leptin Adiponectin PPAR-γ	↑ Leptin vs. water ↓ Adiponectin, PPAR-γ vs. water	
Finamor, 2021 [28]	Swiss Mice	*N* = 12 (6/6)	12 weeks	80 mg/kg	Water	IL-6 CXCL1 IL-1b IL-18 IL-10	↑ IL-6, CXCL1 (IL-8), IL-1b, IL-18 (mRNA) vs. water ↓ IL-10 (mRNA) vs. water	
Lü, 2022 [45]	C57BL/6J Mice	n.r.	18 weeks	0.5 mg/mL	Control, HFD, HFD + SG	Leptin MCP-1 TNF-α	↑ leptin, TNF-α, MCP-1 vs. SG	
He, 2023 [42]	Sprague Dawley rats	*N* = 96 (24/24/24/24)	1, 2, 4 or 8 weeks	40 mg/kg	Distilled water	TNF-α IL-6 IL-1β IL-4	↑ IL-1β, IL-6, TNF-α in blood vs. water ↑ IL-1β, IL-6, TNF-α in brain vs. water ↑ IL-1β, IL-6, TNF-α (mRNA) in brain vs. water ↑ IL-1β, IL-6, TNF-α in CSF vs. water ↑ IL-4 in serum vs. water ↑ IL-4 in CSF vs. water	IL-4 in brain vs. water
Liu, 2024 [46]	C57BL/6J Mice	*N* = 50 (10/10/10/10/10)	14 days	25, 50, 100 mg/kg	Saline	IL-1 IL-6 TNF-α		IL-1β, IL-6, TNF-α in serum at 100 mg/kg
Zhong, 2024 [30]	C57BL/6 Mice	*N* = 48 (12/12/12/12) *N* = 24 (6/6/6/6)	7 days	40 mg/kg	Distilled water, DSS	TNF-α IL-6 IL-1β Macrophages Neutrophils	↑ TNF-α, IL-1β, IL-6 in colon (ASP + DSS vs. DSS) ↑ TNF-α, IL-1β, IL-6 in serum (ASP + DSS vs. DSS) ↑ Macrophages and neutrophils in colon (ASP + DSS vs. DSS)	IL-1β, IL-6, TNF-α in colon vs. water IL-1β, IL-6, TNF-α in serum vs. water Macrophages and neutrophils in colon vs. water
Mohammed, 2024 [16]	Sprague Dawley Rats	*N* = 30 (6/6/6/6/6)	12 weeks	40 mg/kg	Saline, Sucrose, Sorbitol	TNF-α IL-6 IL-1 IL-1β IL-10 IFN-γ	↑ TNF-α, IFN-γ, IL-1, IL-6, IL-10, IL-1β vs. all groups	
Ma, 2024 [27]	C57BL/6 Mice	*N* = 40 (10/10/10/10)	4 weeks	80 mg/kg	Saline	IFN-γ TNF-α IL-6 IL-4 IL-10	↑ TNF-α, IFN-γ, IL-6, IL-4 in serum vs. saline ↓ IL-10 in serum vs. saline ↑ TNF-α, IL-6, IFN-γ, IL-4 in the prefrontal cortex and hippocampus vs. saline ↓ IL-10 in the prefrontal cortex and hippocampus vs. saline	
U-pathi, 2024 [29]	Sprague Dawley rats	*N* = 19 (5/7/7)	8 weeks	30 mg/kg (LA), 60 mg/kg (HA)	Water	TNFα IL-6 IL-1β NFκB iNOS IκB	↑ TNF-α, IL-6, IL-1β in cerebral cortex vs. water (HA > LA) ↑ NF-κB, iNOS vs. water (HA > LA) ↓ IκB vs. water (HA > LA)	
Babatunde, 2024 [32]	Wistar rats	*N* = 36 (6 × 6 groups)	14 days	17 mg/kg (LA) and 67 mg/kg (HA)	Water, Sucrose, SCOP	TNF-α IBA1 cells	↑ TNF-α in hippocampus and cortex (ASP + SCOP vs. water) ↑ TNF-α in hippocampus and cortex (SUC + SCOP > ASP + SCOP) ↑ IBA1 in hippocampus and cortex (ASP + SCOP vs. water) ↑ IBA1 in cortex ASP + SCOP > all groups	TNF-α in hippocampus and cortex SCOP + ASP vs. SCOP
Wu, 2025 [24]	Mice C57BL/6J and Monkeys	n.r.	n.r.	0.05%, 0.1% or 0.15%, *p/p*	Sucrose, Water	CX3CL1	↑ CX3CL1 vs. controls	
Farahi, 2025 [33]	BALB/c Mice	*N* = 54 (6 × 9 groups)	2 weeks	400, 2000 mg/kg	Saline	Neutrophils Lymphocytes Monocytes White blood cells IFN-γ IL-4		Neutrophils, lymphocytes, monocytes, and white blood cells at 400 and 2000 mg/kg (*p* > 0.05) IFN-γ and IL-4 vs. saline (*p*-value for IFN-γ = 0.47. *p*-value for IL-4 = 0.21)
Chuang, 2025 [37]	BALB/c Mice	n.r.	3 weeks	0.25 g/L (4 mL/day)	Water	IL-4 IL-5 IL-13 IL-17 IFN-γ NF-κB IκBα	Results in offspring: ↑ IL-4, IL-5, IL-13, IL-17 in lungs vs. water ↓ IFN-γ in lungs vs. water ↑NF-κB vs. water ↓ IκBα in lungs vs. water	
Lawal, 2025 [22]	Wistar rats	*N* = 30 (5 × 6)	14 days	40 mg/kg	Water TT	TNF-α IL-6 IL-1β IL-10	↑ TNF-α, IL-6, IL-1β vs. all groups ↓ IL-10 vs. all groups	

Abbreviations: ↑, significant increase; ↓, significant decrease; APM, ASP-treated group; ASP, Aspartame; ADI, Acceptable Daily Intake; BALB/c, Inbred albino mouse strain; C57BL/6J, Inbred mouse strain; CSF, Cerebrospinal fluid; CX3CL1, Chemokine involved in neuron-glia communication; CXCL1, Chemokine ligand 1, DSS, Dextran sulfate sodium; HFD, High fat diet; HA/LA High Aspartame/Low Aspartame dose groups; Hsp70, Heat shock protein 70; IFN-γ, Interferon gamma; IBA1, Ionized calcium binding adaptor molecule 1; IκB, Inhibitor of nuclear factor kappa B; IL-1/IL-1β, Interleukin-1 beta; IL-2/IL-4/IL-5/IL-6/IL-10/IL-13/IL-17, interleukins; iNOS/nNOS, Inducible/Neuronal nitric oxide synthase; JNK3,c-Jun N-terminal kinase 3; MCP-1, Monocyte chemoattractant protein-1; MTX, Methotrexate; NF-κB Nuclear factor kappa-light-chain-enhancer of activated B cells; PPAR-γ; Peroxisome proliferator-activated receptor gamma; SCOP, Scopolamine; SG, Siraitia grosvenorii (natural sweetener); TNF-α, Tumor necrosis factor alpha. * Variations with *p* < 0.05 were considered to be significant.

**Table 4:** Results of in vivo studies assessing the impact of sucralose consumption on inflammation markers (by chronological order).

Study	Animal Type	Sample Size	Time Exposure	Dose Exposure	Control Type	Outcomes	Significant * Variations in Inflammation Markers Between the Initiation and Final Timepoints	Non-Significant Variations in Inflammation Markers Between the Initiation and Final Timepoints
Bian, 2017 [5]	C57BL/6 Mice	20 (10/10)	6 months	0.1 mg/mL (5 mg/kg/day)	Water	MMP-2 iNOS TNF-α IL-6 MMP-9 IL-1β	↑ MMP-2, iNOS (mRNA) in the liver vs. water	TNF-α, IL-6, MMP-9, IL-1β (mRNA) in the liver vs. control
Rosales-Gómez, 2018 [15]	CD-1 Mice	72 (8/32/32)	6 weeks, 12 weeks	4.16 mg/mL	Water, Sucrose, Stevia	IL-4 IL-5 IL-10 IFN-γ TNF-α	↓ IFN-γ in Peyer’s patches vs. water/sucrose ↑ TNF-α in Peyer’s patches vs. water/sucrose ↓ IL-4 in Peyer’s patches vs. all groups ↑ IL-5 in Peyer’s patches vs. water ↓ IL-10 in Peyer’s patches vs. all groups ↑ TNF-α in lamina propria vs. water/sucrose ↑ IL-4 in lamina propria vs. all groups	
Martínez-Carrillo, 2019 [25]	CD1 Mice	72 (8 × 9)	6 weeks, 12 weeks	4.1 mg/mL (Splenda^®^, Svetia^®^)	Water, Sucrose,	TNF-α IL-6 IL-17 CD8^+^ CD4^+^ CD3^+^	↑ CD8^+^, IL-6 and IL-17 in Peyer’s patches vs. water/sucrose ↑ CD4^+^, IL-6 and IL-17 in lamina propria vs. water/sucrose ↓ CD8^+^ in lamina propria vs. water/sucrose	CD3^+^ in lamina propria vs. water/sucrose TNF-α vs. water/sucrose
Sánchez-Tapia, 2019 [51]	Wistar rats	108 (18 × 6)	4 months	1.5%	Water, Sucrose, Glucose, Honey, Fructose, Brown sugar	TNF-α TLR4 Myd88 JNK NF-kB	↑ TLR4, Myd88, JNK, TNF-α in adipose tissue vs. water	NF-kB in adipose tissue vs. water
Farid, 2020 [48]	BALB/c albino mice	80 (16 × 5)	8 weeks, 16 weeks	5.2 mg/mL	Water, Sucrose, Stevia	LPS IL-10 IL-8 IL-6	↑ LPS, IL-6, IL-8 vs. water ↓ IL-10 vs. water	
Sánchez-Tapia, 2020 [50]	Wistar rats	108 (18 × 6)	4 months	1.5% in water	Water, Sucrose, Glucose, Honey, Fructose, Brown sugar	TLR-4 TLR-2 NF-kB	↑ TLR4, TLR-2, NF-kB in colon	
Li, 2020 [39]	C57BL/6 mice	n.r.	36 days	1.5 mg/mL	Water, AOM/DSS	TNFα IL-1β IL-6 IL-10 TLR4 Myd88 NF-κB TRAF6 IκBα	↑ TNF-α, TLR4 (mRNA) in colon vs. water ↑ TNF-α, IL-1β (mRNA) in colon (AOM/DSS + SUC vs. AOM/DSS) ↓ IL-10, TRAF6 (mRNA) in colon (AOM/DSS + SUC vs. AOM/DSS) ↑ TNF-α, TLR4, MyD88 in colon vs. water ↓ IL-10, IκBα in colon vs. water ↑ TNF-α, TLR4, MyD88 in colon (AOM/DSS + SUC vs. AOM/DSS) ↓ IL-10, IκBα, TRAF6 in colon (AOM/DSS + SUC vs. AOM/DSS)	
Dai, 2020 [47]	C57BL/6 Mice	n.r.	6 weeks	5–15 mg/kg	Water	TNF-α IFN-γ IL-1β IL-6	↑ IL-1β, IFN-γ, TNF-α (mRNA) in colon vs. water ↑ TNF-α, IL-1β, IL-6 in colon vs. water ↑ IL-6, TNF-α (mRNA) in liver vs. water	
Dai, 2021 [49]	C57BL/6 Mice	8 (then 12/12 pups)	6 weeks	5 mg/kg	Water	IL-6 IL-1β TNFα IFN-γ	↑ IL-1β, TNF-α (mRNA) in small intestine vs. water	IL-6, IFN-γ (mRNA) in small intestine vs. water
Guo, 2021 [17]	C57BL/6 Mice	24 (6/6/6/6)	6 weeks	1.5 mg/mL	Water, DSS	TLR5 MyD88 NF-κB TNFα IL1-β IL-10 IL-18 IL-17a IL-22 NLRP6 NLRP3 NLRP12	↑ NF-κB, MyD88, TLR5 (mRNA) in colon vs. water ↑ IL-22, ↓ IL-10, ↓ NLRP12 in colon vs. water ↑ TNFα, IL1-β, IL-18, IL-17a, IL-22, NLRP3 in colon (SUC + DSS vs. DSS) ↓ IL-10, NLRP12 in colon (SUC + DSS vs. DSS)	
Escoto, 2021 [44]	CD1 Mice	54 (6/24/24)	6–12 weeks	4.16 mg/mL	Sucrose, Stevia	TGF-β IL-12 IL-17	↑ IL-12, IL-17 vs. control ↓ TGF-β vs. control	
Zhang, 2022 [34]	C57BL/6 Mice	50 (10/10/10/10/10)	28 days	5 mg/kg	Water, DSS	TNF-α IL-6 IL-17A	↑ IL-6, IL-17A, TNF-α (mRNA) in colonic tissue (DSS + SUC vs. water) ↓ TNF-α, IL-17A, IL-6 (mRNA) in colonic tissue (DSS + SUC vs. DSS) ↑ IL-6, IL-17A, TNF-α in colonic tissue (DSS + SUC vs. water) ↓ TNF-α, IL-17A, IL-6 in colonic tissue (DSS + SUC vs. DSS)	
Madbouly, 2022 [36]	BALB/c Albino Mice	100 (5 × 20)	8 weeks, 16 weeks	0.3 mg/mL	Water, SAC- cyclamate mixture	TNF-α IL-6 LPS	↑ IL-6 vs. water ↑ LPS vs. water ↑ LPS vs. SAC- cyclamate	TNF-α vs. water TNF-α, IL-6 vs. SAC- cyclamate
Yang, 2024 [23]	C57BL/6 Mice	21 (7/7/7)	12 weeks	0.1 mg/mL	Water	LPS IL-6	↑ LPS and IL-6 vs. control	
Mohammed, 2024 [16]	Sprague Dawley Rats	*N* = 30 (6/6/6/6/6)	12 weeks	5 mg/kg	Saline, Sucrose, Sorbitol	TNF-α IL-6 IL-1 IL-1β IL-10 IFN-γ	↑ TNF-α, IFN-γ, IL-1, IL-6, IL-10, IL-1β vs. controls	
Hanawa, 2021 [35]	C57BL/6J Mice	n.r.	8 weeks	150 mg/kg	Water	TNF-α IFN-γ IL1-β MAdCAM-1 GLP1R GLP2R		IFN-γ, IL-1β, and TNF-α vs. control

Abbreviations: ↑, significant increase; ↓, significant decrease. AOM, Azoxymethane; BALB/c, Inbred albino mouse strain; C57BL/6/C57BL/6J, Common inbred mouse strain; CD-1, Outbred mouse strain; CD3^+^CD8^+^, cytotoxic T cells; DSS, Dextran sodium sulfate; IFN-γ, Interferon gamma; IL-1/IL-1β/IL-4/IL-5/IL-6/IL-8/IL-10/IL-12/IL-17/IL-17A, Interleukins; iNOS, Inducible nitric oxide synthase; IκBα, Inhibitor of NF-κB; LPS, Lipopolysaccharide; MMP-2/MMP-9, Matrix metalloproteinases, MyD88 Adaptor protein in immune signaling via TLR pathways; NF-κB, Nuclear factor kappa-light-chain-enhancer of activated B cells; n.r., Not reported; Splenda^®^, Svetia^®^, Stevia, Commercial sweeteners; SUC, sucralose; TLR4, Toll-like receptor 4; TNF-α, Tumor necrosis factor alpha; TRAF6, TNF receptor-associated factor 6; TGF-β, Transforming growth factor beta. * Variations with *p* < 0.05 were considered to be significant.

**Table 5:** Results of in vivo studies assessing the impact of Ace-K consumption on inflammation markers (by chronological order).

Study	Animal Type	Sample Size	Time Exposure	Dose Exposure	Control Type	Outcomes	Significant * Variations in Inflammation Markers Between the Initiation and Final Timepoints	Non-Significant Variations in Inflammation Markers Between the Initiation and Final Timepoints
Zhai, 2024 [43]	C57BL/6 Mice, zebrafish	15 (5/5/5) 36 (12/12/12)	28 days 21 days	21 mg/L, 7 mg/L, 10 μg/L, 100 μg/L	Water	Inflammatory cells Mucin Transcriptomic analyses	↑ infiltration of inflammatory Cells in colon vs. water ↓ secretion of protective Mucin in colon vs. water ↑ inflammation, destruction to the crypt and epithelial cells, depletion of mucus ↑ genes related to cytokine-cytokine receptor, chemokine signaling, and IBD	
Bridge-Comer, 2023 [41]	C57Bl/6 Mice	36 (6/6/6/6/6/6)	11 weeks offspring	12.5 mM ACK in drinking water	Water, Fructose	TNF-α IL1b Nlrp3 Vegfa Tgfb Pparg	↑ TNF-α (mRNA) female offspring in skin Tissue vs. water, fructose	IL-1b, Nlrp3, Vegfa, Tgfb, Pparg (mRNA) female offspring in skin Tissue vs. water, fructose TNF-α, IL-1b, Nlrp3, Vegfa, Tgfb, Pparg (mRNA) male offspring in skin Tissue vs. water, fructose
Lin, 2021 [26]	ApoE-/- Mice	36 (9/9/9/9)	8 weeks	15 mg/kg	Saline	TNF-α Ccl2 IL-6		TNF-α, Ccl2, IL-6 (mRNA) vs. control
Hanawa, 2021 [35]	C57BL/6J Mice	n.r.	8 weeks	150 mg/kg	Water	TNF-α IFN-γ IL1-β MAdCAM-1	↑ IFN-γ, IL-1β, and TNF-α, MAdCAM-1 (mRNA) in small intestinal mucosa vs. water	
Shou, 2024 [52]	C57BL/6 Mice	24 (8/8/8)	11 weeks	40 mg/kg, 120 mg/kg	Water	TNF-α IL-6 IL-1β LPS	↑ TNFα, IL-6 in high dose vs. water ↑ LPS vs. water	IL-6 in low dose vs. water IL-1β vs. water

Abbreviations: ↑, significant increase; ↓, significant decrease; Ace-K, acesulfame potassium; ApoE-/-, genetically modified mice lacking ApoE gene; C57BL/6/C57BL/6J, Common inbred mouse strain used; Ccl2, Chemokine (C–C motif) ligand 2; IL-6, Interleukin-6; IFN-γ, Interferon gamma; IBD, Inflammatory Bowel Disease; LPS, Lipopolysaccharide; MAdCAM-1, Mucosal vascular addressing cell adhesion molecule 1; n.r., Not reported; Pparg, peroxisome proliferator-activated receptor gamma; Tgfb, Transforming growth factor beta; TNF-α, Tumor necrosis factor alpha; Vegfa, Vascular endothelial growth factor A; vs., versus. * Variations with *p* < 0.05 were considered to be significant.

**Table 6:** Results of in vivo studies assessing the impact of saccharin consumption on inflammation markers.

Study	Animal Type	Sample Size	Time Exposure	Dose Exposure	Control Type	Outcomes	Significant * Variations in Inflammation Markers Between the Initiation and Final Timepoints	Non-Significant Variations in Inflammation Markers Between the Initiation and Final Timepoints
Zhang, 2022 [34]	C57BL/6 mice	50 (10/10/10/10/10)	28 days	5 mg/kg	Water, DSS	TNF-α IL-6 IL-17A	↑ IL-6, IL-17A, TNF-α (mRNA) in colonic tissue (DSS + SAC vs. water) ↓ TNF-α, IL-17A, IL-6 (mRNA) in colonic tissue (DSS + SAC vs. DSS) ↑ IL-6, IL-17A, TNF-α in colonic tissue (DSS + SAC vs. water) ↓ TNF-α, IL-17A, IL-6 in colonic tissue (DSS + SAC vs. DSS)	
Hanawa, 2021 [35]	C57BL/6J mice	n.r.	8 weeks	50 mg/kg	Water	TNF-α IFN-γ IL1-β		IFN-γ, IL-1β, and TNF-α vs. control
Bian, 2017 [5]	C57BL/6J mice	20 (10/10)	6 months	0.3 mg/mL	Water	iNOS TNF-α IL-6 IL-1β	↑ iNOS & TNF-α (mRNA) vs. control	IL-1β, IL-6 vs. control
Madbouly, 2022 [36]	BALB/c albino mice	100 (5 × 20)	8 weeks, 16 weeks	20 mg/mL	Water SUC	TNF-α IL-6 LPS	↑ IL-6 vs. water ↑ TNF-α vs. water ↑ LPS vs. controls	TNF-α vs. SUC IL-6 vs. SUC

Abbreviations: ↑, significant increase; ↓, significant decrease; IL, interleukin; LPS, lipopolysaccharide; TNF-α, Tumor necrosis factor alpha. * Effects with *p* < 0.05 were considered to be significant.

## Supplementary Materials

The following supporting information can be downloaded at: https://www.mdpi.com/article/10.3390/nu17203251/s1. Table S1: Prisma checklist; Table S2: Search strategies; Table S3: Quality assessment according ARRIVE Guidelines; Table S4: Quality assessment according SYRCLE tool.

## References

[1] Prescott M.P., Ben Hassen T., El Bilali H. (2023). Editorial: Obesity and Sustainability. Front. Nutr..

[2] Russell C., Baker P., Grimes C., Lindberg R., Lawrence M.A. (2023). Global Trends in Added Sugars and Non-Nutritive Sweetener Use in the Packaged Food Supply: Drivers and Implications for Public Health. Public Health Nutr..

[3] Kossiva L., Kakleas K., Christodouli F., Soldatou A., Karanasios S., Karavanaki K. (2024). Chronic Use of Artificial Sweeteners: Pros and Cons. Nutrients.

[4] Harris E. (2023). WHO Warns Against Artificial Sugars for Weight Loss. JAMA.

[5] Bian X., Tu P., Chi L., Gao B., Ru H., Lu K. (2017). Saccharin Induced Liver Inflammation in Mice by Altering the Gut Microbiota and Its Metabolic Functions. Food Chem. Toxicol..

[6] Bian X., Chi L., Gao B., Tu P., Ru H., Lu K. (2017). Gut Microbiome Response to Sucralose and Its Potential Role in Inducing Liver Inflammation in Mice. Front. Physiol..

[7] Khanna D., Khanna S., Khanna P., Kahar P., Patel B.M. (2022). Obesity: A Chronic Low-Grade Inflammation and Its Markers. Cureus.

[8] Magali R.-L., Jason M., World Health Organization (2022). Health Effects of the Use of Non-Sugar Sweeteners: A Systematic Review and Meta-Analysis. World Health Organization. https://iris.who.int/handle/10665/353064.

[9] Debras C., Deschasaux-Tanguy M., Chazelas E., Sellem L., Druesne-Pecollo N., Esseddik Y., Szabo de Edelenyi F., Agaësse C., De Sa A., Lutchia R. (2023). Artificial Sweeteners and Risk of Type 2 Diabetes in the Prospective NutriNet-Santé Cohort. Diabetes Care.

[10] Mohan V., Manasa V.S., Abirami K., Unnikrishnan R., Gayathri R., Geetha G., RamyaBai M., Padmavathi S., Rajalakshmi M., Pradeepa R. (2024). Effect of Replacing Sucrose in Beverages with Nonnutritive Sweetener Sucralose on Cardiometabolic Risk Factors Among Asian Indian Adults with Type 2 Diabetes: A 12-Week Randomized Controlled Trial. Diabetes Ther..

[11] Scott C.E., Stamataki N., Harrold J.A., Raben A., Halford J.C.G. (2025). Health Impact Database Development for Sweeteners and Sweetness Enhancers: The SWEET Project. Nutr. Bull..

[12] Gauthier E., Milagro F.I., Navas-Carretero S. (2024). Effect of low-and non-calorie sweeteners on the gut microbiota: A review of clinical trials and cross-sectional studies. Nutrition.

[13] Fan Y., Pedersen O. (2021). Gut microbiota in human metabolic health and disease. Nat. Rev. Microbiol..

[14] Pang M.D., Kjølbæk L., Bastings J.J.A.J., Andersen S.S.H., Umanets A., Sost M.M., Navas-Carretero S., Reppas K., Finlayson G., Hodgkins C.E. (2025). Effect of sweeteners and sweetness enhancers on weight management and gut microbiota composition in individuals with overweight or obesity: The SWEET study. Nat. Metab..

[15] Rosales-Gómez C.A., Martínez-Carrillo B.E., Reséndiz-Albor A.A., Ramírez-Durán N., Valdés-Ramos R., Mondragón-Velásquez T., Escoto-Herrera J.A. (2018). Chronic Consumption of Sweeteners and Its Effect on Glycaemia, Cytokines, Hormones, and Lymphocytes of GALT in CD1 Mice. BioMed Res. Int..

[16] Mohammed D.M., Abdelgawad M.A., Ghoneim M.M., Alhossan A., Al-Serwi R.H., Farouk A. (2024). Impact of Some Natural and Artificial Sweeteners Consumption on Different Hormonal Levels and Inflammatory Cytokines in Male Rats: In Vivo and In Silico Studies. ACS Omega.

[17] Guo M., Liu X., Tan Y., Kang F., Zhu X., Fan X., Wang C., Wang R., Liu Y., Qin X. (2021). Sucralose Enhances the Susceptibility to Dextran Sulfate Sodium (DSS) Induced Colitis in Mice with Changes in Gut Microbiota. Food Funct..

[18] Page M.J., McKenzie J.E., Bossuyt P.M., Boutron I., Hoffmann T.C., Mulrow C.D., Shamseer L., Tetzlaff J.M., Akl E.A., Brennan S.E. (2021). The PRISMA 2020 Statement: An Updated Guideline for Reporting Systematic Reviews. BMJ.

[19] Percie Du Sert N., Hurst V., Ahluwalia A., Alam S., Avey M.T., Baker M., Browne W.J., Clark A., Cuthill I.C., Dirnagl U. (2020). The ARRIVE Guidelines 2.0: Updated Guidelines for Reporting Animal Research. PLoS Biol..

[20] Hooijmans C.R., Rovers M.M., De Vries R.B., Leenaars M., Ritskes-Hoitinga M., Langendam M.W. (2014). SYRCLE’s Risk of Bias Tool for Animal Studies. BMC Med. Res. Methodol..

[21] Abdel-Salam O.M.E., Salem N.A., Hussein J.S. (2012). Effect of Aspartame on Oxidative Stress and Monoamine Neurotransmitter Levels in Lipopolysaccharide-Treated Mice. Neurotox. Res..

[22] Lawal A.O., Agboola O.O., Akinjiyan M.O., Ijatuyi T.T., Dahunsi D.T., Okeowo O.M., Folorunso I.M., Olajuyigbe O.J., Elekofehinti O.O. (2025). The Antioxidative, Anti-Inflammatory and Anti-Apoptotic Effects of Tetrapleura Tetraptera (Aidan) Ethanol Leaf Extract in the Brain of Wistar Rats Exposed to Aspartame. Mol. Neurobiol..

[23] Yang L., Wang S., Jin J., Wang J., Chen W., Xue Y., Sheng L., Zhai Y., Yao W. (2024). Sucralose Triggers Insulin Resistance Leading to Follicular Dysplasia in Mice. Reprod. Toxicol..

[24] Wu W., Sui W., Chen S., Guo Z., Jing X., Wang X., Wang Q., Yu X., Xiong W., Ji J. (2025). Sweetener Aspartame Aggravates Atherosclerosis through Insulin-Triggered Inflammation. Cell Metab..

[25] Martínez-Carrillo B.E., Rosales-Gómez C.A., Ramírez-Durán N., Reséndiz-Albor A.A., Escoto-Herrera J.A., Mondragón-Velásquez T., Valdés-Ramos R., Castillo-Cardiel A. (2019). Effect of Chronic Consumption of Sweeteners on Microbiota and Immunity in the Small Intestine of Young Mice. Int. J. Food Sci..

[26] Lin C.-H., Li H.-Y., Wang S.-H., Chen Y.-H., Chen Y.-C., Wu H.-T. (2021). Consumption of Non-Nutritive Sweetener, Acesulfame Potassium Exacerbates Atherosclerosis through Dysregulation of Lipid Metabolism in ApoE^−/−^ Mice. Nutrients.

[27] Ma H., Deng J., Liu J., Jin X., Yang J. (2024). Daytime Aspartame Intake Results in Larger Influences on Body Weight, Serum Corticosterone Level, Serum/Cerebral Cytokines Levels and Depressive-like Behaviors in Mice than Nighttime Intake. NeuroToxicology.

[28] Finamor I.A., Bressan C.A., Torres-Cuevas I., Rius-Pérez S., Da Veiga M., Rocha M.I., Pavanato M.A., Pérez S. (2021). Long-Term Aspartame Administration Leads to Fibrosis, Inflammasome Activation, and Gluconeogenesis Impairment in the Liver of Mice. Biology.

[29] U-pathi J., Yeh Y.-C., Chen C.-W., Owaga E.E., Hsieh R.-H. (2023). Relationship between Aspartame-Induced Cerebral Cortex Injury and Oxidative Stress, Inflammation, Mitochondrial Dysfunction, and Apoptosis in Sprague Dawley Rats. Antioxidants.

[30] Zhong M., Li L., Liu W., Wen W., Ma L., Jin X., Li G., Yang J. (2024). Acceptable Daily Intake of Aspartame Aggravates Enteritis Pathology and Systemic Inflammation in Colitis Mouse Model. J. Food Sci..

[31] Lebda M.A., Tohamy H.G., El-Sayed Y.S. (2017). Long-Term Soft Drink and Aspartame Intake Induces Hepatic Damage via Dysregulation of Adipocytokines and Alteration of the Lipid Profile and Antioxidant Status. Nutr. Res..

[32] Babatunde O.O., Christiana A.B., Sunday O.I., Philemon A.O., Tolulope A.S., Meashack I.O., Ganiyu O. (2024). Comparative Effect of Selected Caloric and Non-Caloric Sweeteners on Some Neuroinflammatory Indices in Brain Cortex and Hippocampus of Scopolamine-Induced Rat. Nutrire.

[33] Farahi S.M.M., Forouzanfar F., Memar B., Rashidi R., Mahdipour R., Riahi-Zanjani B., Sadeghi M. (2025). Aspartame Subacute Exposure Does Not Affect Immune System of BALB/c Mice Following a Tiered Approach. Tissue Cell.

[34] Zhang X., Gu J., Zhao C., Hu Y., Zhang B., Wang J., Lv H., Ji X., Wang S. (2022). Sweeteners Maintain Epithelial Barrier Function Through the miR-15b/RECK/MMP-9 Axis, Remodel Microbial Homeostasis, and Attenuate Dextran Sodium Sulfate-Induced Colitis in Mice. J. Agric. Food Chem..

[35] Hanawa Y., Higashiyama M., Kurihara C., Tanemoto R., Ito S., Mizoguchi A., Nishii S., Wada A., Inaba K., Sugihara N. (2021). Acesulfame Potassium Induces Dysbiosis and Intestinal Injury with Enhanced Lymphocyte Migration to Intestinal Mucosa. J. Gastroenterol. Hepatol..

[36] Madbouly N., El-Hadad G., El Amir A., Farid A. (2022). Hazard Effects of Chronic Consumption of Sucralose and Saccharin-Sodium Cyclamate Mixture in Murine Model. Egypt. J. Chem..

[37] Chuang H.-C., Yang Y.-C.S.H., Chou H.-C., Chen C.-M. (2025). Maternal Aspartame Exposure Alters Lung Th1/Th2 Cytokine Balance in Offspring through Nuclear Factor-κB Activation. Int. Immunopharmacol..

[38] Ashok I., Sheeladevi R. (2015). Oxidant Stress Evoked Damage in Rat Hepatocyte Leading to Triggered Nitric Oxide Synthase (NOS) Levels on Long Term Consumption of Aspartame. J. Food Drug Anal..

[39] Li X., Liu Y., Wang Y., Li X., Liu X., Guo M., Tan Y., Qin X., Wang X., Jiang M. (2020). Sucralose Promotes Colitis-Associated Colorectal Cancer Risk in a Murine Model Along with Changes in Microbiota. Front. Oncol..

[40] Choudhary A.K., Sheela Devi R. (2015). Longer Period of Oral Administration of Aspartame on Cytokine Response in Wistar Albino Rats. Endocrinol. Nutr..

[41] Bridge-Comer P.E., Vickers M.H., Ferraro S., Pagnon A., Reynolds C.M., Sigaudo-Roussel D. (2023). Maternal Intake of Either Fructose or the Artificial Sweetener Acesulfame-K Results in Differential and Sex-Specific Alterations in Markers of Skin Inflammation and Wound Healing Responsiveness in Mouse Offspring: A Pilot Study. Nutrients.

[42] He X., Zhong Q., Yan K., Li G., Yang J. (2023). Oral Exposure to an Acceptable Daily Intake Dose of Aspartame Induced a Delayed Proinflammatory Cytokine Response in the Cerebrospinal Fluid of Rats. Food Chem. Toxicol..

[43] Zhai Z., Zhang Y., Liang X., Li J., Chen Z., Zhang J., Li W., Wang T., He Q., Li F. (2024). Acesulfame Potassium Triggers Inflammatory Bowel Disease via the Inhibition of Focal Adhesion Pathway. J. Hazard. Mater..

[44] Escoto J.A., Martínez-Carrillo B.E., Ramírez-Durán N., Ramírez-Saad H., Aguirre-Garrido J.F., Valdés-Ramos R. (2021). Consumo crónico de edulcorantes en ratones y su efecto sobre el sistema inmunitario y la microbiota del intestino delgado. Biomédica.

[45] Lü K., Song X., Zhang P., Zhao W., Zhang N., Yang F., Guan W., Liu J., Huang H., Ho C.-T. (2022). Effects of Siraitia Grosvenorii Extracts on High Fat Diet-Induced Obese Mice: A Comparison with Artificial Sweetener Aspartame. Food Sci. Hum. Wellness.

[46] Liu Q.K. (2024). Mechanisms of Action and Therapeutic Applications of GLP-1 and Dual GIP/GLP-1 Receptor Agonists. Front. Endocrinol..

[47] Dai X., Guo Z., Chen D., Li L., Song X., Liu T., Jin G., Li Y., Liu Y., Ajiguli A. (2020). Maternal Sucralose Intake Alters Gut Microbiota of Offspring and Exacerbates Hepatic Steatosis in Adulthood. Gut Microbes.

[48] Farid A., Hesham M., El-Dewak M., Amin A. (2020). The hidden hazardous effects of stevia and sucralose consumption in male and female albino mice in comparison to sucrose. Saudi Pharm. J..

[49] Dai X., Wang C., Guo Z., Li Y., Liu T., Jin G., Wang S., Wang B., Jiang K., Cao H. (2021). Maternal sucralose exposure induces Paneth cell defects and exacerbates gut dysbiosis of progeny mice. Food Funct..

[50] Sánchez-Tapia M., Miller A.W., Granados-Portillo O., Tovar A.R., Torres N. (2020). The Development of Metabolic Endotoxemia Is Dependent on the Type of Sweetener and the Presence of Saturated Fat in the Diet. Gut Microbes.

[51] Sánchez-Tapia M., Martínez-Medina J., Tovar A.R., Torres N. (2019). Natural and Artificial Sweeteners and High Fat Diet Modify Differential Taste Receptors, Insulin, and TLR4-Mediated Inflammatory Pathways in Adipose Tissues of Rats. Nutrients.

[52] Shou N., Rensing C., Lin Q., Xu W., Fu K., Yuan X., Wu D., Wang F., Li Y., Shi Z. (2024). Acesulfame Potassium Induces Hepatic Inflammation and Fatty Acids Accumulation via Disturbance of Carnitine Metabolism and Gut Microbiota. Food Biosci..

[53] Riboli E., Beland F.A., Lachenmeier D.W., Marques M.M., Phillips D.H., Schernhammer E., Afghan A., Assunção R., Caderni G., Corton J.C. (2023). Carcinogenicity of Aspartame, Methyleugenol, and Isoeugenol. Lancet Oncol..

[54] Huwart S.J.P., Fayt C., Gangarossa G., Luquet S., Cani P.D., Everard A. (2024). TLR4-Dependent Neuroinflammation Mediates LPS-Driven Food-Reward Alterations during High-Fat Exposure. J. Neuroinflamm..

[55] Baird I.M., Shephard N.W., Merritt R.J., Hildick-Smith G. (2000). Repeated Dose Study of Sucralose Tolerance in Human Subjects. Food Chem. Toxicol. Int. J. Publ. Br. Ind. Biol. Res. Assoc..

[56] Schiffman S.S., Rother K.I. (2013). Sucralose, a Synthetic Organochlorine Sweetener: Overview of Biological Issues. J. Toxicol. Environ. Health B Crit. Rev..

[57] Aguayo-Guerrero J.A., Méndez-García L.A., Solleiro-Villavicencio H., Viurcos-Sanabria R., Escobedo G. (2024). Sucralose: From Sweet Success to Metabolic Controversies-Unraveling the Global Health Implications of a Pervasive Non-Caloric Artificial Sweetener. Life.

[58] Castle L., Andreassen M., Aquilina G., Bastos M.L., Boon P., Fallico B., FitzGerald R., Frutos Fernandez M.J., Grasl-Kraupp B., EFSA Panel on Food Additives and Flavourings (FAF) (2025). Re-Evaluation of Acesulfame K (E 950) as Food Additive. EFSA J. Eur. Food Saf. Auth..

[59] Castle L., Andreassen M., Aquilina G., Bastos M.L., Boon P., Fallico B., FitzGerald R., Frutos Fernandez M.J., Grasl-Kraupp B., EFSA Panel on Food Additives and Flavourings (FAF) (2024). Re-Evaluation of Saccharin and Its Sodium, Potassium and Calcium Salts (E 954) as Food Additives. EFSA J. Eur. Food Saf. Auth..

[60] Sylvetsky A.C., Mitchell E.L., Grilo M.F., Um C.Y., Wang Y., Hodge R.A., Patel A.V., McCullough M.L. (2025). Cross-Sectional Associations between Consumption of Non-Nutritive Sweeteners and Diet Quality among United States Adults in the Cancer Prevention Study-3. Am. J. Clin. Nutr..

[61] Miller P.E., Perez V. (2014). Low-calorie sweeteners and body weight and composition: A meta-analysis of randomized controlled trials and prospective cohort studies. Am. J. Clin. Nutr..

[62] McGlynn N.D., Khan T.A., Wang L., Zhang R., Chiavaroli L., Au-Yeung F., Lee J.J., Noronha J.C., Comelli E.M., Blanco Mejia S. (2022). Association of Low- and No-Calorie Sweetened Beverages as a Replacement for Sugar-Sweetened Beverages With Body Weight and Cardiometabolic Risk: A Systematic Review and Meta-analysis. JAMA Netw. Open.

[63] Fitch S.E., Payne L.E., van de Ligt J.L.G., Doepker C., Handu D., Cohen S.M., Anyangwe N., Wikoff D. (2021). Use of acceptable daily intake (ADI) as a health-based benchmark in nutrition research studies that consider the safety of low-calorie sweeteners: A systematic map. BMC Public Health.

[64] Knezovic Z., Jurcevic Zidar B., Pribisalic A., Luetic S., Jurcic K., Knezovic N., Sutlovic D. (2025). Artificial Sweeteners in Food Products: Concentration Analysis, Label Practices, and Cumulative Intake Assessment in Croatia. Nutrients.

