# Supplementary material for: Impact of Artificial Sweeteners on Inflammation Markers: A Systematic Review of Animal Studies
Source: Nutrients. 2025 Oct 16;17(20):3251. doi: 10.3390/nu17203251 (PMC12567400; doi:10.3390/nu17203251)
Supplement: Supplementary file 1 [file nutrients-17-03251-s001.zip › Table S4 suppl data SYRCLE checklist.pdf]

Table S4. Quality assessment according SYRCLE tool

Yes = Low risk of bias; No = High risk of bias; Unclear = Risk of bias not assessable due to insufficient information

First Author, Year of Publication	1. Sequence generation	2. Baseline characteristics	3. Allocation concealment	4. Random housing	5. Blinding	6. Random outcome assessment	7. Blinding	8. Incomplete outcome data	9. Selective outcome reporting	10. Other source of bias
**Liu, 2025**	Unclear	Yes	Unclear	Unclear	Unclear	Unclear	Unclear	Yes	Yes	Yes
**Farahi, 2025**	No	Yes	Unclear	No	Unclear	Unclear	Unclear	Yes	Yes	Yes
**Zhai, 2024**	Unclear	Yes	Unclear	Unclear	Unclear	Unclear	Unclear	Yes	Yes	Yes
**Zhong, 2024**	Unclear	Yes	Unclear	Unclear	Unclear	Unclear	Unclear	Yes	Yes	Yes
**Mohammed, 2024**	No	Yes	Unclear	No	Unclear	Unclear	Unclear	Yes	Yes	Yes
**Ma, 2024**	Unclear	Yes	Unclear	Unclear	Unclear	Unclear	Unclear	Yes	Yes	Yes
**U-pathi, 2023**	Unclear	Yes	Unclear	No	Unclear	Unclear	Unclear	Yes	Yes	Yes
**He, 2023**	Unclear	Yes	Unclear	Unclear	Unclear	Unclear	Unclear	Yes	Yes	Yes
**Bridge-Comer, 2023**	Unclear	Yes	Unclear	Unclear	Unclear	Unclear	Unclear	Yes	Yes	Yes
**Zhang, 2022**	Unclear	Yes	Unclear	No	Unclear	Unclear	Unclear	Yes	Yes	Yes
**Finamor, 2021**	No	Yes	Unclear	No	Unclear	Unclear	Unclear	Yes	Yes	Yes
**Hanawa, 2021**	No	Yes	Unclear	No	Unclear	Unclear	Unclear	Yes	Yes	Yes
**Sánchez-Tapia, 2020**	No	Yes	Unclear	No	Unclear	Unclear	Unclear	Yes	Yes	Yes
**Farid, 2020**	No	Yes	Unclear	No	Unclear	Unclear	Unclear	Yes	Yes	Yes
**Sánchez-Tapia, 2019**	Unclear	Yes	Unclear	Unclear	Unclear	Unclear	Unclear	Yes	Yes	Yes
**Bian, 2017**	Unclear	Yes	Unclear	Unclear	Unclear	Unclear	Unclear	Yes	Yes	Yes
**Mart-nez-Carrillo, 2019**	No	Yes	Unclear	No	Unclear	Unclear	Unclear	Yes	Yes	Yes
**Rosales-Gómez, 2019**	Unclear	Yes	Unclear	Unclear	Unclear	Unclear	Unclear	Yes	Yes	Yes
**Ashok, 2015**	No	Yes	Unclear	No	Unclear	Unclear	Unclear	Yes	Yes	Yes
**Bian, 2017**	Unclear	Yes	Unclear	Unclear	Unclear	Unclear	Unclear	Yes	Yes	Yes
**Lebda, 2017**	Unclear	Yes	Unclear	Unclear	Unclear	Unclear	Unclear	Yes	Yes	Yes

**Madbouly, 2022**	Unclear	Yes	Unclear	Unclear	Unclear	Unclear	Unclear	Yes	Yes	Yes
**Abdel-Salam, 2012**	Unclear	Yes	Unclear	Unclear	Unclear	Unclear	Unclear	Yes	Yes	Yes
**Lin, 2021**	Unclear	Yes	Unclear	Unclear	Unclear	Unclear	Unclear	Yes	Yes	Yes
**Babatunde,2024**	Unclear	Yes	Unclear	Unclear	Unclear	Unclear	Unclear	Yes	Yes	Yes
**Escoto, 2021**	No	Yes	Unclear	No	Unclear	Unclear	Unclear	Yes	Yes	Yes
**Chuang, 2025**	Unclear	Yes	Unclear	Unclear	Unclear	Unclear	Unclear	Yes	Yes	Yes
**Graneri 2021**	Unclear	Yes	Unclear	Unclear	Unclear	Unclear	Unclear	Yes	Yes	Yes
**Lawal 2025**	No	Yes	Unclear	No	Unclear	Unclear	Unclear	Yes	Yes	Yes
**Wu 2025**	Unclear	Yes	Unclear	Unclear	Unclear	Unclear	Unclear	Yes	Yes	Yes
**Li 2020**	No	Yes	Unclear	No	Unclear	Unclear	Unclear	Yes	Yes	Yes
**Shou 2024**	Unclear	Yes	Unclear	Unclear	Unclear	Unclear	Unclear	Yes	Yes	Yes
**Dai 2020**	Unclear	Yes	Unclear	Unclear	Unclear	Unclear	Unclear	Yes	Yes	Yes
**Lü 2022**	Unclear	Yes	Unclear	Unclear	Unclear	Unclear	Unclear	Yes	Yes	Yes
**Choudhary 2014**	Unclear	Yes	Unclear	Unclear	Unclear	Unclear	Unclear	Yes	Yes	Yes
**Luyao 2024**	Unclear	Yes	Unclear	Unclear	Unclear	Unclear	Unclear	Yes	Yes	Yes
