# Supplementary material for: Impact of Artificial Sweeteners on Inflammation Markers: A Systematic Review of Animal Studies
Source: Nutrients. 2025 Oct 16;17(20):3251. doi: 10.3390/nu17203251 (PMC12567400; doi:10.3390/nu17203251)
Supplement: Supplementary file 1 [file nutrients-17-03251-s001.zip › Table S2 Suppl data search strategy 15 10 2025.pdf]

Table S2. Search strategies

1. Medline

Medline (via Pubmed) was searched 5 May 2025 using keywords (text word) and MeSH Terms.

Set	Search terms	Search type	Results
#1	(artificial AND sweeteners) OR (non AND nutritive AND sweeteners) OR (low AND calorie AND sweeteners AND non AND sugar AND sweetener*) OR aspartame OR sucralose OR saccharin OR (acesulfame AND K) OR (acesulfame AND potassium) OR neotame OR advantame OR cyclamate	advanced	25,255
#2	inflammation OR (inflammation AND mediator*) OR (inflammatory AND pathway*) OR CRP OR (C-reactive AND protein) OR IL-6 OR (interleukin AND 6) OR TNF OR cytokine* OR (NF AND Kb) OR ROS OR (interleukin AND 18) OR IL-18 OR (interleukin AND 1) OR (tumor AND necrosis AND factor) OR (monocyte AND chemoattractant AND protein) OR MCP-1/CCL2 OR E-selectin OR (intercellular AND adhesion AND molecule) OR ICAM-1 OR adiponectin	advanced	1,884,513
#3	#1 AND #2	advanced	1566
#4	Filter: Animals	advanced	802

2. Web of Science

Web of science was searched 5 May 2025 using keywords (text word) in topic**.**

Set	Search terms	Search type	Results
#1	(artificial AND sweeteners) OR (non AND nutritive AND sweeteners) OR (low AND calorie AND sweeteners AND non AND sugar AND sweetener*) OR aspartame OR sucralose OR saccharin OR (acesulfame AND K) OR (acesulfame AND potassium) OR neotame OR advantame OR cyclamate	advanced	13,850
#2	inflammation OR (inflammation AND mediator*) OR (inflammatory AND pathway*) OR CRP OR (C-reactive AND protein) OR IL-6 OR (interleukin AND 6) OR TNF OR cytokine* OR (NF AND Kb) OR ROS OR (interleukin AND 18) OR IL-18 OR (interleukin AND 1) OR (tumor AND necrosis AND factor) OR (monocyte AND chemoattractant AND protein) OR MCP-1/CCL2 OR E-selectin OR (intercellular AND adhesion AND molecule) OR ICAM-1 OR adiponectin	advanced	2,039,367
#3	#1 AND #2	advanced	412

3. Scopus

Scopus was searched 5 May 2025 using keywords.

Set	Search terms	Search type	Results
#1	(artificial AND sweeteners) OR (non AND nutritive AND sweeteners) OR (low AND calorie AND sweeteners AND non AND sugar AND sweetener*) OR aspartame OR sucralose OR saccharin OR (acesulfame AND K) OR (acesulfame AND potassium) OR neotame OR advantame OR cyclamate	advanced	66,889

#2	inflammation OR (inflammation AND mediator*) OR (inflammatory AND pathway*) OR CRP OR (C-reactive AND protein) OR IL-6 OR (interleukin AND 6) OR TNF OR cytokine* OR (NF AND Kb) OR ROS OR (interleukin AND 18) OR IL-18 OR (interleukin AND 1) OR (tumor AND necrosis AND factor) OR (monocyte AND chemoattractant AND protein) OR MCP-1/CCL2 OR E-selectin OR (intercellular AND adhesion AND molecule) OR ICAM-1 OR adiponectin	advanced	369,665
#3	#1 AND #2	advanced	673
#4	Limit to: animals	advanced	369
