# Supplementary material for: Impact of Artificial Sweeteners on Inflammation Markers: A Systematic Review of Animal Studies
Source: Nutrients. 2025 Oct 16;17(20):3251. doi: 10.3390/nu17203251 (PMC12567400; doi:10.3390/nu17203251)
Supplement: Supplementary file 1 [file nutrients-17-03251-s001.zip › Table S3 ARRIVE guidelines checklist.pdf]

Table S3. Quality assessment according ARRIVE guidelines

First Author, Year of Publication	1. Study Design	2. Sample Size	3. Inclusion and exclusion criteria	4. Randomisation	5. Blinding	6. Outcome measures	7. Statistical methods	8. Experimental animals	9. Experimental procedures	10. Results	11. Abstrac	12. Background	13. Objectives	14. Ethical statement	15. Housing and husbandry	16. Animal care and monitoring	17. Interpretation/scientific implications	18. Generalisability/translation	19. Protocol registration	20. Data access	21. Declaration of interest
Liu, 2025	+	?	-	?	?	+	+	+	+	+	+	+	+	+	+	?	+	+	-	+	+
Farahi, 2025	+	?	+	+	+	+	+	+	+	+	+	+	+	+	+	?	+	+	+	+	+
Zhai, 2024	+	?	?	?	?	+	+	+	+	+	+	+	+	+	+	?	+	+	-	+	+
Zhong, 2024	+	?	-	+	?	+	+	+	+	+	+	+	+	+	+	?	+	+	-	+	+
Mohammed, 2024	+	?	?	?	?	+	+	+	+	+	+	+	+	+	+	?	+	+	-	+	+
Ma, 2024	+	?	+	+	?	+	+	+	+	+	+	+	+	+	+	+	+	+	-	+	+
U-pathi, 2023	+	?	?	+	?	+	+	+	+	+	+	+	+	+	+	?	+	+	-	+	+
He, 2023	+	?	?	?	?	+	+	+	+	+	+	+	+	+	+	?	+	+	-	+	+
Bridge-Comer, 2023	+	?	?	?	?	+	+	+	+	+	+	+	+	+	+	?	+	?	-	+	+
Zhang, 2022	+	?	?	?	+	+	+	+	+	+	+	+	+	+	+	?	+	+	-	+	+
Finamor, 2021	+	?	?	-	-	+	+	+	+	+	+	+	+	+	+	?	+	+	-	+	+

**Hanawa, 2021**	+	?	?	-	-	+	+	+	+	+	+	+	+	+	-	?	+	+	-	-	+
**Sánchez-Tapia, 2020**	+	+	?	-	-	+	+	+	+	+	+	+	+	+	+	?	+	+	-	-	+
**Farid, 2020**	+	?	?	-	?	+	+	+	+	+	+	+	+	+	+	?	+	+	-	-	+
**Sánchez-Tapia, 2019**	+	?	?	?	?	+	+	+	+	+	+	+	+	+	+	?	+	+	-	?	+
**Bian, 2017**	+	?	?	-	?	+	+	+	+	+	+	+	+	+	+	+	+	+	-	+	+
**Mart-nez-Carrillo, 2019**	+	?	+	-	?	+	+	+	+	+	+	+	+	+	+	?	+	+	-	+	+
**Rosales-Gómez, 2019**	+	?	?	?	-	+	+	+	+	+	+	+	+	+	-	?	+	+	-	+	+
**Ashok, 2015**	+	?	?	-	?	+	+	+	+	+	+	+	+	+	?	?	+	+	-	+	+
**Bian, 2017**	+	?	?	?	?	+	+	+	+	+	+	+	+	+	+	?	+	+	-	+	+
**Lebda, 2017**	+	+	?	?	?	+	+	+	+	+	+	+	+	+	?	?	+	+	-	+	+
**Madbouly, 2022**	+	?	?	?	?	+	+	+	+	+	+	+	+	+	+	?	+	+	-	+	+
**Abdel-Salam, 2012**	+	?	?	?	?	+	+	+	+	+	+	+	+	+	+	?	+	+	-	+	+

**Lin, 2021**	+	?	?	?	?	+	+	+	+	+	+	+	+	+	+	?	+	+	-	+	+
**Babatunde, 2024**	+	?	?	?	?	+	+	+	+	+	+	+	+	+	+	?	+	+	-	+	+
**Escoto, 2021**	+	?	?	-	?	+	+	+	+	+	+	+	+	+	+	?	+	?	-	-	+
**Chuang, 2025**	+	?	?	?	?	+	+	+	+	+	+	+	+	+	+	?	+	+	-	+	+
**Graneri 2021**	+	?	?	?	?	+	+	+	+	+	+	+	+	+	+	?	+	+	-	+	+
**Lawal 2025**	+	?	?	-	?	+	+	+	+	+	+	+	+	+	+	?	+	+	-	-	+
**Wu 2025**	+	?	?	?	?	+	+	+	+	+	+	+	+	+	+	?	+	+	-	+	+
**Li 2020**	+	?	?	-	?	+	+	+	+	+	+	+	+	+	?	?	+	+	-	+	+
**Shou 2024**	+	?	?	?	?	+	+	+	+	+	+	+	+	+	+	?	+	+	-	+	+
**Dai 2020**	+	?	?	?	?	+	+	+	+	+	+	+	+	+	+	?	+	+	-	+	+
**Lü 2022**	+	?	?	?	?	+	+	+	+	+	+	+	+	+	+	?	+	+	-	+	+
**Choudhary 2014**	+	?	?	?	?	+	+	+	+	+	+	+	+	+	+	?	+	+	-	?	+
**Luyao 2024**	+	?	?	?	?	+	+	+	+	+	+	+	+	+	+	?	+	+	-	?	+
